# A report of three cases of patients with tubulointerstitial nephritis with IgM-positive plasma cells, treatment, and serum-IgM as a sensitive marker for relapse

**DOI:** 10.1186/s12882-023-03253-8

**Published:** 2023-07-04

**Authors:** Ryota Akagi, Akira Ishii, Keiichi Kaneko, Naoya Kondo, Hideki Yokoi, Takeshi Matsubara, Sachiko Minamiguchi, Yoshihiko Kanno, Motoko Yanagita

**Affiliations:** 1 Department of Nephrology, Graduate School of Medicine, Kyoto University, 54 Shogoin Kawahara-Cho, Sakyo-Ku, Kyoto, 606-8507 Japan; 2 Department of Nephrology, Kyoto Katsura Hospital, 17 Yamadahirao-Cho, Nishikyo-Ku, Kyoto, 615-8257 Japan; 3 Department of Diagnostic Pathology, Graduate School of Medicine, Kyoto University, 54 Shogoin Kawahara-Cho, Sakyo-Ku, Kyoto, 606-8507 Japan; 4 Department of Nephrology, Tokyo Medical University, 6-7-1 Nishi-Shinjuku, Shinjuku-Ku, Tokyo, 160-0023 Japan; 5 Institute for the Advanced Study of Human Biology (ASHBi), Kyoto University, Yoshida Konoe-Cho, Sakyo-Ku, Kyoto, 606-8501 Japan

**Keywords:** Tubulointerstitial nephritis with IgM-positive plasma cells, Relapse, Serum IgM, Distal renal tubular acidosis (d-RTA), Fanconi syndrome, Sjögren syndrome

## Abstract

**Background:**

Tubulointerstitial nephritis with IgM-positive plasma cells (IgMPC-TIN) is a newer disease about which there are many unclear points. Glucocorticoid therapy is effective in many cases of IgMPC-TIN; however, relapse during glucocorticoid tapering has been reported. Relapse and its treatment are poorly defined.

**Case Presentation:**

Case 1 was a 61-year-old man with renal dysfunction and proteinuria. Tubulointerstitial nephritis and IgM-positive plasma cells were observed in a renal biopsy. He was diagnosed with IgMPC-TIN accompanied by Fanconi syndrome and distal renal tubular acidosis (d-RTA). Prednisolone (PSL; 30 mg daily, 0.45 mg/kg/day) treatment was highly effective, and PSL was gradually tapered and discontinued after 1 year. However, 1 month after PSL discontinuation, therapeutic markers were elevated. Therefore, PSL (10 mg daily, 0.15 mg/kg/day) was administered, and the markers indicated improvement.

Case 2 was a 43-year-old woman referred for renal dysfunction and proteinuria. Laboratory data revealed that she had primary biliary cholangitis (PBC), d-RTA, and Fanconi syndrome. A renal biopsy showed accumulation of IgM-positive plasma cells in the tubulointerstitium without any glomerular changes. A diagnosis of IgMPC-TIN was made and the patient was started on PSL (35 mg daily, 0.6 mg/kg/day). Therapeutic markers decreased immediately and PSL was discontinued after 1 year. Three months later, the proteinuria and Fanconi syndrome worsened. PSL treatment was restarted (20 mg daily, 0.35 mg/kg/day) and markers indicated improvement.

Case 3 was a 45-year-old woman with renal dysfunction and proteinuria. Tubulointerstitial nephritis and IgM-positive plasma cells were observed in a renal biopsy. The patient had PBC, Sjögren syndrome, d-RTA, and Fanconi syndrome, and the diagnosis of IgMPC-TIN was made. The patient was started on PSL (30 mg daily, 0.4 mg/kg/day) and disease markers decreased immediately. However, when PSL was tapered to 15 mg daily (0.2 mg/kg/day), the patient’s serum IgM levels increased; therefore, we maintained the PSL at 15 mg daily (0.2 mg/kg/day).

**Conclusion:**

We report three cases of relapsed IgMPC-TIN associated with reduction or discontinuation of glucocorticoid therapy. In these cases, elevation of serum IgM preceded that of other markers such as urinary β_2_-microglobulin, proteinuria, and glycosuria. We recommend monitoring serum IgM levels while tapering glucocorticoids; a maintenance dose of glucocorticoid should be considered if relapse is suspected or anticipated.

## Background

Various causes of tubulointerstitial nephritis (TIN) have been identified, including infectious and drug-related causes, as well as autoimmune causes such as Sjögren syndrome, sarcoidosis, and IgG4-related diseases [1]. However, in many cases of TIN, the cause has not been identified. TIN with IgM-positive plasma cells (IgMPC-TIN) is a relatively new disease first described in 2017 [2]. Although its pathophysiological mechanisms are still largely unknown, the major clinical features of IgMPC-TIN include high serum IgM (s-IgM) levels; high prevalence (> 80%) of distal renal tubular acidosis (d-RTA) [2, 3], Fanconi syndrome and positive anti-mitochondrial antibodies (AMA); and complications of primary biliary cholangitis (PBC) (46%) [2, 4] or Sjӧgren syndrome (31%) [2, 5]. Pathologically, IgMPC-TIN is characterized by accumulation of IgM-positive plasma cells (identified using IgM/CD138 immunohistochemistry) within the interstitium, whereas conventional TIN is characterized by infiltration with IgG-positive cells. In most cases of IgMPC-TIN, intermediate-dose glucocorticoid therapy is highly effective, but there are a number of reports of patients relapsing during glucocorticoid tapering [6–8]. There is no clear definition of relapse or consensus of when treatment should be intensified. Minato et al. reported that cyclosporine A or mizoribine in combination with prednisolone (PSL) was effective in controlling the disease in the case of relapse [7], but the efficacy and method of administration of immunosuppressants in relapse cases have not been established.

Here, we report three patients with IgMPC-TIN and relapse during glucocorticoid tapering. In these cases, elevation of s-IgM level preceded that of other disease activity markers, including urinary β_2_-microglobulin (u-β_2_MG), proteinuria, and glycosuria. Thus, s-IgM may be a useful marker for detecting disease relapse.

## Case Presentations

### Case 1

**Fig. 1:**
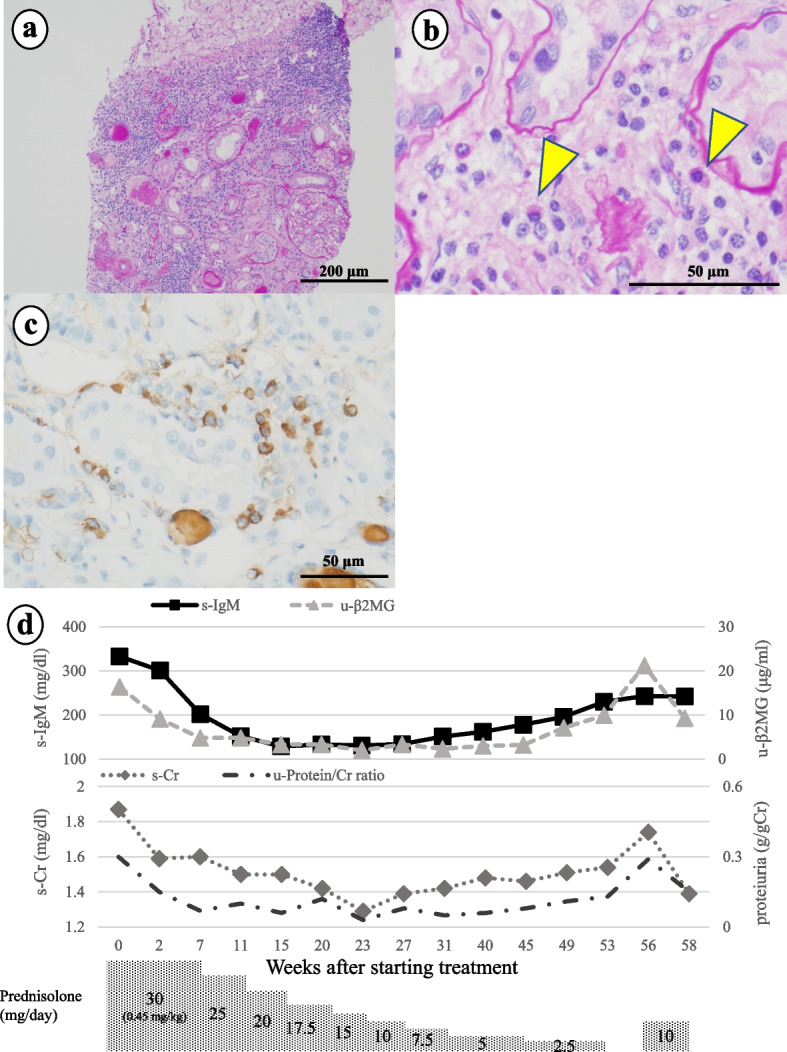
The renal biopsy specimen in case 1 contained 21 glomeruli, 4 with global sclerosis. **a** Periodic acid-Schiff (PAS) stain (100 ×) showed inflammatory cell infiltration in tubules and interstitium. **b** PAS stain (400 ×) showed infiltration of lymphocytes and plasma cells (yellow arrows) in interstitium. **c** Immunohistochemistry for IgM (400 ×) suggested existing IgM-positive plasma cells in tubules and interstitium. In three high-power fields (HPFs), the average number of IgM-PCs per HPF was 22. **d** Clinical course in case 1: After the patient started prednisolone (PSL; 30 mg daily; 0.45 mg/kg/day), markers decreased immediately, and we tapered and discontinued PSL as shown here. However, 1 month after PSL discontinuation, serum IgM (s-IgM), serum creatinine (s-Cr), urinary β2-microglobulin (u-β_2_MG), and proteinuria levels were elevated. Therefore, PSL 10 mg daily (0.15 mg/kg/day) was administered, and these markers improved

A 61-year-old man was admitted to our department because of renal dysfunction and proteinuria. He had a history of rheumatoid arthritis treated with methotrexate. Laboratory examination revealed elevations in serum creatinine (s-Cr; 1.54 mg/dL), s-IgM (333 mg/dL), and AMA-M2 antibody (144 U/mL). Serum potassium (3.4 mEq/L), phosphorus (2.8 mg/dL), and uric acid (2.0 mg/dL) levels were low. Urinalysis showed proteinuria (0.4 g/day), glycosuria (4.0 g/day), panamino-aciduria, and elevated u-β_2_MG (19.6 μg/mL). Venous blood gas analysis revealed normal anion gap metabolic acidosis with alkaline urinary pH, which were suggestive of d-RTA. On renal biopsy, lymphocytes and plasma cells showed diffuse infiltration in the tubules and interstitium (Fig. 1a, b). Immunofluorescence staining was negative for immunoglobulins and complement. IgM and plasma cell immunohistochemistry in the tubules and interstitium were positive. An average of 22 IgM-positive plasma cells (IgM-PCs) per high-power field (HPF) were observed in three HPFs (Fig. 1c). The patient was diagnosed with IgMPC-TIN accompanied by Fanconi syndrome and d-RTA. After starting the patient on PSL (30 mg daily: 0.45 mg/kg/day), levels of s-IgM, urine protein, and u-β_2_MG decreased immediately, and PSL was gradually tapered and discontinued after 1 year. However, 1 month after PSL discontinuation, s-IgM was elevated again before re-elevation of s-Cr, u-β_2_MG, and urine protein levels. Therefore, PSL (10 mg daily: 0.15 mg/kg/day) was re-administered, and levels of these markers indicated improvement (Fig. 1d).

### Case 2

**Fig. 2:**
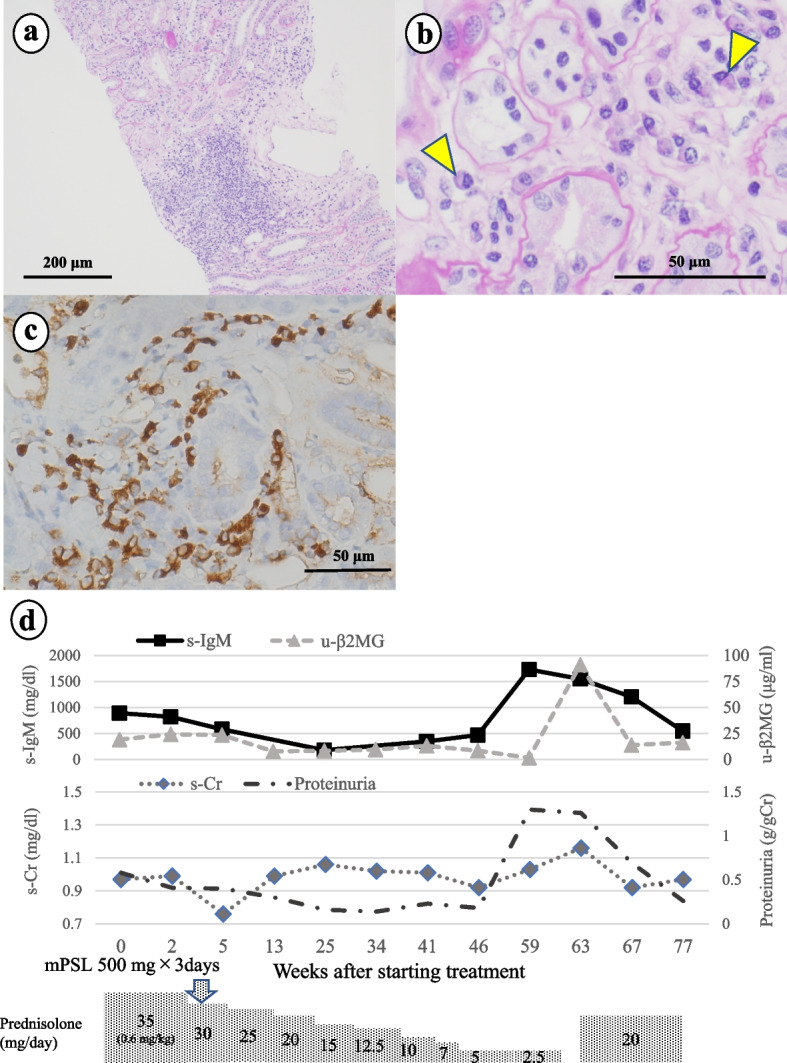
The renal biopsy specimen in case 2 contained 43 glomeruli, with no major glomerular changes except three with global sclerosis. **a** Periodic acid-Schiff (PAS) stain (100 ×) showed inflammatory cell infiltrate in tubules and interstitium. **b** PAS stain (400 ×) showed infiltration of lymphocytes and plasma cells (yellow arrows) in interstitium. **c** Immunohistochemistry for IgM (400 ×) suggested the presence of IgM-positive plasma cells in tubules and interstitium. The average number of IgM-PCs in three HPFs was 32. **d** Clinical course in case 2: After the patient received methylprednisolone (mPSL; 500 mg daily) intravenously for three consecutive days, she started on prednisolone (PSL; 35 mg daily; 0.6 mg/kg/day), and disease markers decreased immediately. Thus, we gradually tapered and discontinued PSL treatment as shown here. Three months after PSL discontinuation, serum IgM, proteinuria, and urinary β2-microglobulin (u-β_2_MG) levels increased. Therefore, PSL 20 mg daily (0.35 mg/kg/day) was administered and disease markers improved

A 43-year-old woman was referred to our department because of renal dysfunction and proteinuria. She had a history of PBC treated with ursodeoxycholic acid. Laboratory test results showed renal dysfunction (s-Cr: 0.98 mg/dL, eGFR: 54.7 mL/min/1.73 m^2^), s-IgM (948 mg/dL), and hepatobiliary enzyme levels, and decreased serum potassium (3.3 mEq/L), phosphorus (2.5 mg/dL), and uric acid (2.2 mg/dL) levels. The patient was positive for AMA-M2 antibodies (289 U/mL). Urinalysis findings were as follows: proteinuria (0.5 g/day), glycosuria (10.1 g/day), pan-aminoaciduria, and u-β_2_MG (20.3 μg/mL). Venous blood gas analysis revealed normal anion gap metabolic acidosis with alkaline urinary pH. Renal biopsy showed diffuse lymphocyte and plasma cell infiltration into the tubules and interstitium (Fig. 2a, b). Immunofluorescence staining was negative for immunoglobulins and complement. IgM and plasma cell immunohistochemistry in the tubules and interstitium were positive, with an average of 32 IgM-PCs in three HPFs (Fig. 2c). Therefore, the patient was diagnosed with IgMPC-TIN accompanied by Fanconi syndrome, d-RTA, and PBC. Methylprednisolone (mPSL, 500 mg daily, intravenously) was started for three consecutive days, and dosing continued with PSL (35 mg daily: 0.6 mg/kg/day). Levels of s-IgM, urine protein, and u-β_2_MG decreased immediately. Treatment with PSL was gradually tapered and discontinued after 1 year. However, 3 months after PSL discontinuation, s-IgM increased prior to re-elevation of urine protein and hepatobiliary enzyme levels, and symptoms of Fanconi syndrome worsened (Fig. 2d). Therefore, PSL (20 mg daily: 0.35 mg/kg/day) was re-administered, and the markers indicated improvement.

### Case 3

**Fig. 3:**
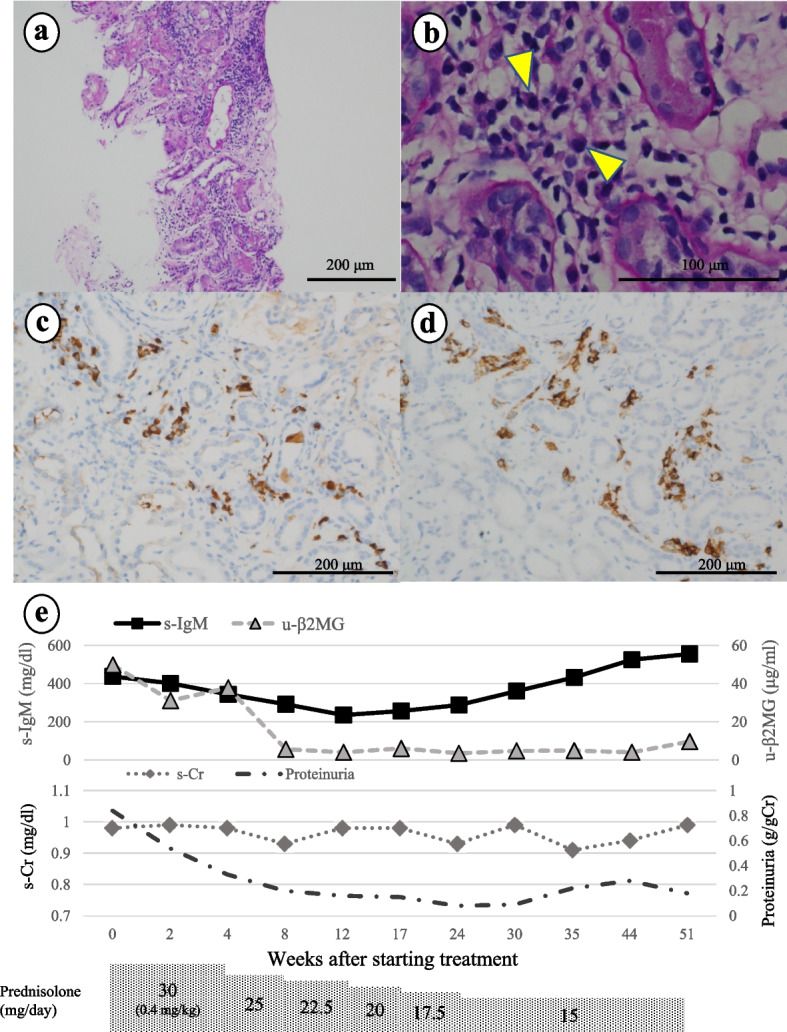
The renal biopsy specimen from case 3 contained 39 glomeruli, 5 with global sclerosis but no major glomerular changes. **a** Periodic acid-Schiff (PAS) stain (100 ×), showed inflammatory cell infiltrate in tubules and interstitium. **b** PAS stain (400 ×) revealed that the inflammatory cells included lymphocytes and plasma cells (yellow arrows). Immunohistochemistry for (**c**) IgM (200 ×), and (**d**) CD38 (200 ×) revealed the presence of many IgM-positive plasma cells in tubules and interstitium. The average number of IgM-PCs per HPF in three HPFs was 13. **e** Clinical course in case 3: After the patient started on treatment with prednisolone (PSL 30 mg daily: 0.4 mg/kg/day) for 4 weeks, markers decreased immediately. We gradually tapered PSL to 15 mg daily (0.2 mg/kg/day) as shown here; however, serum IgM levels increased again, and PSL was continued at 15 mg daily (0.2 mg/kg/day)

A 45-year-old woman was admitted with renal dysfunction and proteinuria. Laboratory examination showed elevated s-Cr (increasing from 0.55 to 1.06 mg/dL in 5 years), s-IgM (449 mg/dL), biliary enzyme levels, and AMA-M2 antibodies (57.6 U/mL). Serum potassium (3.4 mEq/L), phosphorus (2.8 mg/dL), and uric acid (2.0 mg/dL) levels were low. Urinalysis showed proteinuria (1.0 g/day), glycosuria (7.5 g/day), pan-aminoaciduria, and elevated u-β_2_MG levels (31.7 μg/mL). Venous blood gas analysis revealed normal anion gap metabolic acidosis with alkaline urinary pH. Renal biopsy results showed severe lymphocyte and plasma cell infiltration in the tubules, and moderate interstitial fibrosis was observed (Fig. 3a, b). Although immunofluorescence staining was negative for immunoglobulins and complement, immunohistochemistry for IgM/CD38 was positive for plasma cells in the tubules and interstitium (Fig. 3c, d). On average, there were 13 IgM-PCs in three HPFs. Therefore, we diagnosed the patient with IgMPC-TIN accompanied by Fanconi syndrome, d-RTA, PBC, and Sjӧgren syndrome (the patient met the criteria for Sjӧgren syndrome, including a positive Saxon test result and inflammatory cell infiltration in the salivary duct following a lip biopsy). After starting PSL (30 mg daily: 0.4 mg/kg/day for 4 weeks), the patient’s s-IgM, proteinuria, glycosuria, and u-β_2_MG levels decreased immediately. PSL was gradually tapered to 15 mg daily (0.2 mg/kg/day) 6 months after starting treatment. However, s-IgM level increased to 566 mg/dL prior to re-elevation of proteinuria, glycosuria, and u-β_2_MG. Therefore, we continued treatment with PSL at 15 mg daily (0.2 mg/kg/day) (Fig. 3e).

## Discussion and conclusions

Here, we report three cases of relapse following diagnosis and treatment of IgMPC-TIN. Pathologically, this disease is characterized by infiltration of IgM-positive plasma cells, identified by IgM/CD138 immunohistochemistry, in the interstitium, whereas conventional TIN is mainly infiltrated by IgG-positive plasma cells. Before Takahashi et al. proposed IgMPC-TIN in 2017, cases of TIN complicated with PBC had been reported [9–15]. Although its pathophysiological mechanisms are still largely unknown, the major clinical features of IgMPC-TIN are high s-IgM levels; high prevalence (> 80%) of d-RTA, Fanconi syndrome and positivity for AMA; and complication of PBC (46%) or Sjӧgren syndrome (31%) [2]. In some cases of IgMPC-TIN, liver biopsies have shown that IgM-PCs infiltrate in the portal tract [6, 8], which suggests that IgM-positive plasma cells themselves may be responsible for major syndromes such as TIN, dRTA, Fanconi syndrome, and PBC.

Although initial glucocorticoid therapy is reported to be effective [2], there is no clear definition of relapse or consensus around treating a recurrence. To the best of our knowledge, there are only four case reports of relapse; in three of the four cases, s-IgM and u-β_2_MG trends during the glucocorticoid taper were described in detail [6–8]. The clinical features of six patients with relapse, including our three patients, are described below. At initial presentation, the mean age was 52 ± 7 years, two were men and four were women, s-Cr was 1.32 ± 0.28 mg/dL, proteinuria was 0.5 ± 0.2 g/gCr, s-IgM was 627 ± 199 mg/dL, and u-β_2_MG was 45.5 ± 23.6 μg/mL. All patients were treated with PSL, and the initial dose was 36 ± 7 mg/day. At the time of relapse, s-Cr was 1.34 ± 0.30 mg/dL, s-IgM was 858 ± 555 mg/dL, and PSL had been discontinued in four of six patients. The period from treatment initiation to relapse was 12 ± 4 months.

Matsuoka-Uchiyama et al. reported that proteinuria, glycosuria, u-β_2_MG, and s-IgM are useful markers for detecting relapse [6]. In all three of our patients, s-IgM increased prior to re-elevation of u-β_2_MG, urine protein levels, and renal dysfunction. Thus, we consider that s-IgM may be the most sensitive marker for IgMPC-TIN relapse. In the renal biopsies, there were 34 ± 26 IgM-PCs/HPF in five relapsed patients, including the previously reported cases, and 65 ± 30 IgM-PCs/HPF in 11 patients without relapse who were taking glucocorticoids, which was not significantly different by t-test (*p* = 0.09). Serum IgM at the first visit was 935 ± 428 mg/dL in non-relapsed patients and 627 ± 199 mg/dL in relapsed patients, which was not significantly different by t-test (*p* = 0.07). Our results indicate that we should pay attention to relapse, regardless of the s-IgM level at initial presentation or the number of IgM-PCs in renal biopsy.

Additionally, careful glucocorticoid tapering is required to prevent relapse: four of the six relapses, including previously reported cases, occurred after glucocorticoid therapy discontinuation. In relapse cases of IgMPC-TIN, a maintenance glucocorticoid dose may be required. However, the adverse effects associated with long-term administration of glucocorticoid should be considered [16]. One of our patients (case 3) is overeating and becoming obese as a side effect of PSL treatment; we are considering reducing the PSL dose and adding immunosuppressants. Minato et al. reported a good response after treatment with add-on cyclosporine A or mizoribine [7]. In cases of long-term glucocorticoid use, the addition of immunosuppressants such as cyclosporine A or mizoribine may be considered, although further data to support this approach are needed.

In conclusion, we propose that s-IgM may be the most sensitive marker for relapse of IgMPC-TIN; elevation of s-IgM preceded changes in other markers such as u-β_2_MG, proteinuria, and glycosuria in our three cases. We recommend carefully tapering the glucocorticoid dose while monitoring s-IgM levels. A maintenance glucocorticoid dose should be considered if relapse is suspected or anticipated. In addition, we recommend close monitoring for relapse regardless of the s-IgM level at initial presentation or the number of IgM-PCs in a renal biopsy.

## Data Availability

The dataset supporting the conclusions of this article is included within the article.

## Abbreviations

s-Cr: Serum creatinine
IgM: Immunoglobulin M
s-IgM: Serum IgM
u-β_2_MG: Urinary β_2_-microglobulin
PBC: Primary biliary cholangitis
d-RTA: Distal renal tubular acidosis
PSL: Prednisolone; mPSL, methylprednisolone
IgM-PCs: IgM-positive plasma cells
HPF: High-power field
TIN: Tubulointerstitial nephritis
IgMPC-TIN: Tubulointerstitial nephritis with IgM-positive plasma cells
